# Efficacy and safety evaluation of type‐3 phosphodiesterase inhibitor Cilostazol in mild cognitive impairment

**DOI:** 10.1002/alz.086323

**Published:** 2025-01-09

**Authors:** Satoshi Saito, Haruko Yamamoto, Masanori Fukushima, Masafumi Ihara, the COMCID Trial Investigator Group

**Affiliations:** ^1^ National Cerebral and Cardiovascular Center, Suita, Osaka Japan; ^2^ Foundation of Learning Health Society Institute, Nagoya, Aichi Japan

## Abstract

**Background:**

Cilostazol, a selective type‐3 phosphodiesterase inhibitor, ameliorates β‐amyloid accumulation by facilitating intramural periarterial drainage.

**Method:**

Patients with mild cognitive impairment were registered in the COMCID study, an investigator‐initiated, double‐blinded, multi‐center, phase‐II clinical trial. The primary endpoint was the Mini‐Mental State Examination score. Several psychological assessments and circulating β‐amyloid‐albumin complex levels were evaluated as secondary and exploratory endpoints.

**Result:**

Patients were randomly allocated to the Cilostazol (n = 82) or placebo (n = 84) group. There were no serious adverse events caused by the drug intervention. The primary and secondary efficacy endpoints were not different between the two groups. A significant negative correlation between the blood levels of β‐amyloid‐albumin complexes at baseline and the 24‐week visit was observed in the Cilostazol group (*P* = .005) but not in the placebo group (*P* = .336).

**Conclusion:**

Cilostazol was well‐tolerated and may have facilitated β‐amyloid clearance from the brain to the blood but did not prevent cognitive decline.

